# Validation of Suitable Housekeeping Genes for the Normalization of mRNA Expression for Studying Tumor Acidosis

**DOI:** 10.3390/ijms19102930

**Published:** 2018-09-26

**Authors:** Silvia Lemma, Sofia Avnet, Michael Joseph Meade, Tokuhiro Chano, Nicola Baldini

**Affiliations:** 1 Orthopaedic Pathophysiology and Regenerative Medicine Unit, Istituto Ortopedico Rizzoli IRCCS, 40136 Bologna, Italy; silvia.lemma@ior.it (S.L.); sofia.avnet@ior.it (S.A.); Michael.Meade@stonybrookmedicine.edu (M.J.M.); 2 Department of Clinical Laboratory Medicine, Shiga University of Medical Science, Otsu, Shiga 520-2192, Japan; chano@belle.shiga-med.ac.jp; 3 Department of Biomedical and Neuromotor Sciences, University of Bologna, 40123 Bologna, Italy

**Keywords:** acidosis, housekeeping gene, tumor microenvironment, RT-qPCR

## Abstract

Similar to other types of cancer, acidification of tumor microenvironment is an important feature of osteosarcoma, and a major source of cellular stress that triggers cancer aggressiveness, drug resistance, and progression. Among the different effects of low extracellular pH on tumor cells, we have recently found that short-term exposure to acidosis strongly affects gene expression. This alteration might also occur for the most commonly used housekeeping genes (HKG), thereby causing erroneous interpretation of RT-qPCR data. On this basis, by using osteosarcoma cells cultured at different pH values, we aimed to identify the ideal HKG to be considered in studies on tumor-associated acidosis. We verified the stability of 15 commonly used HKG through five algorithms (NormFinder, geNorm, BestKeeper, ΔCT, coefficient of variation) and found that no universal HKG is suitable, since at least four HKG are necessary for proper normalization. Furthermore, according to the acceptable range of values, *YWHAZ*, *GAPDH*, *GUSB*, and *18S rRNA* were the most stable reference genes at different pH. Our results will be helpful for future investigations focusing on the effect of altered microenvironment on cancer behavior, particularly on the effectiveness of anticancer therapies in acid conditions.

## 1. Introduction

Human tumors survive adverse microenvironments that derive from uncontrolled cell proliferation and anarchic tissue organization [1]. In the last years, among the different features of the adverse and altered tumor microenvironment, extracellular acidosis has been a major field of investigation as it is a direct cause of cancer progression and therapeutic resistance [2]. Intratumoral acidosis arises from the Warburg phenotype: an increased glucose metabolism [3] that causes high secretion of protons and a high excretion of lactic acid [4,5] which combines with poor perfusion and elevated activity and/or expression of proton pumps [2,6], ultimately resulting in an extracellular pH between 6.5 and 6.9 [7].

Osteosarcoma is the most common primary malignancy of bone, affecting children and young adults [8,9]. As for other solid malignancies, we have recently shown that acidosis modulates osteosarcoma behavior, fosters cellular metabolic switch, epigenetic modifications, and other cellular alterations that result in a more aggressive tumor phenotype characterized by increased cancer stemness, drug resistance, and invasiveness [10,11,12,13,14,15,16]. Current management of osteosarcoma patients includes surgical resection and intensive chemotherapy [17]. Unfortunately, current treatment strategies are often inadequate at eradicating the disease, with outcomes plateauing over the past decades and five-year survival standing at under 60–70% [18]. There is thus still an urgent need to improve the knowledge about the underlying mechanisms regulating cancer aggressiveness under low extracellular pH conditions, and gene expression analysis can provide critical information in this field. Real-time quantitative reverse transcription polymerase chain reaction (RT-qPCR) is a widely used method to measure gene expression at gene transcription level. In RT-qPCR, quantification of specific messenger RNA (mRNA) is obtained through the comparison with the expression of endogenous controls, that is, the housekeeping genes (HKG). The choice of HKG is critical. By definition, HKG expression is constant and should not be affected by experimental conditions [19]. However, HKG are often blindly adopted from the literature and used across several experimental conditions. Specific stimuli may affect their expression and, if unrecognized, these unexpected changes could result in erroneous evaluation of the expression of genes of interest [20]. Interstitial acidosis is a known regulator of genetic and epigenetic modification [15,21] and an important stress for tumor cell. Low extracellular pH might thus be included among the different stimuli that can cause HKG instability.

In this study, by using osteosarcoma cells, we investigated the influence of extracellular acidosis on the stability of the most widely adopted HKG in order to identify HKG that can be reliably used for gene expression analysis. The ultimate aim is the identification of the best reference genes for the normalization of RT-qPCR assay to obtain consistent and accurate expression data in studies addressing the characterization of the effects of intratumoral acidosis on cancer behavior and recurrence, or the effectiveness of anti-cancer therapies.

## 2. Results

### 2.1. Expression Profile of Candidate HKG

We analyzed the expression of 15 previously identified HKG (Table 1; [20]) in three osteosarcoma cell lines (HOS, MG-63, Saos-2) cultured in acid and physiological pH (pH 6.5 and 7.4, respectively).

**Figure 1:**
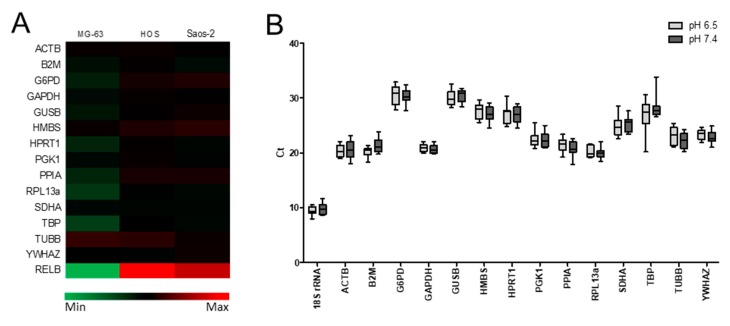
Transcription profiling of the selected HKG. (**A**) Heat map showing the relative expression of the selected genes by deep-sequencing analysis performed on MG-63, HOS, and Saos-2 cell lines cultured under acid pH (pH 6.5) compared to physiological medium (pH 7.4) for 24 h. Colors on the heat map indicate the log_2_ ratios of expression (representing normalized read counts). Green, downregulation; red, upregulation. *RelB* is the positive control of the analysis [15]. (**B**) Box-and-whisker plot indicating range of Cycle threshold (*C*_t_) values of the candidate reference genes in the above mentioned osteosarcoma cells lines in acid or physiological conditions (pH 6.5 and 7.4, respectively). Boxes represent lower and upper quartiles of cycle threshold range with the median indicated as the line across the box; the whiskers represent the 10th and 90th percentiles.

First we verified the purity of the samples by spectrophotometric analysis, and found a A260/280 ratio equal to 2.08 ± 0.005, indicating protein-free pure RNA. Then, we performed the deep sequencing analysis of 15 HKG by Illumina Genome Analyzer sequencing, revealing a stable transcriptome profile for most of the selected HKG (Figure 1A), as expected for candidate HKG, with a slight fluctuation of the expression of some genes, such as *TBP*, *TUBB*, or *RPL13a*.

To compare the mRNA transcription levels of HKG, we used the raw threshold cycles (*C*_t_) values. *C*_t_ is inversely proportional to the amount of gene expression [22]. The 15 putative HKG exhibited a broad range of expression, ranging from 9.64 ± 0.91 of *18S rRNA* (highest expression) to 30.39 ± 1.55 of *G6PD* (lower expression; Table 2). Notably, although *18S rRNA* was in general the most expressed HKG, in acidic pH the least expressed gene was *G6PD*, whereas under physiological pH the least expressed gene was *GUSB* (highest *C*_t_ value) (Figure 1, Table 2).

Some HKGs presented different levels of expression depending on the pH conditions. In particular, about half of the HKG were more expressed in acid pH (*18S rRNA*, *ACTB*, *B2M*, *GUSB*, *HPRT1*, *SDHA*, and *TBP*), whereas the other half were less expressed (Table 2). Differences in the level of expression were clear within the same gene between the two pH-culturing conditions. In terms of δ *C*_t_ (Δ*C*_t_), the smallest difference in gene expression was found for *HPRT1* and *PGK1*, whereas the highest difference was detected for *TBP*, *B2M*, and *TUBB* (Table 2). These findings underline the need for an accurate evaluation of HKG stability for an accurate evaluation of gene expression data in acid and physiological pH conditions.

### 2.2. Analysis of the Stability of Candidate Reference Genes in Acid Tumor Microenvironment

The stability of candidate HKG was analyzed through five different statistical methods of assessments: NormFinder [23], geNorm [24], BestKeeper [25], the Δ*C*_t_ method [26], and the evaluation of coefficient of variation [20]. Moreover, we evaluated the minimal number of HKG required for the accurate normalization of RT-qPCR data by performing a pairwise variation (*V_n_*_/*n*+1_) analysis by GeNorm between the normalization factors NF*_n_* and NF*_n_*_+1_. *V* values below the cutoff value 0.15 correspond to the optimal number of genes required for data normalization. The comprehensive ranking of the genes was also evaluated, giving a total of six evaluation methods. The net final rank of the most stable genes was obtained considering the lowest value of the geometric average of the rank obtained from all the algorithms and methods of stability calculation [20]. The smaller the geometric mean, the greater the stability of HKG expression.

First we considered the gene expression of osteosarcoma cell lines cultured under acid pH conditions (pH 6.5; Table 3).

**Figure 2:**
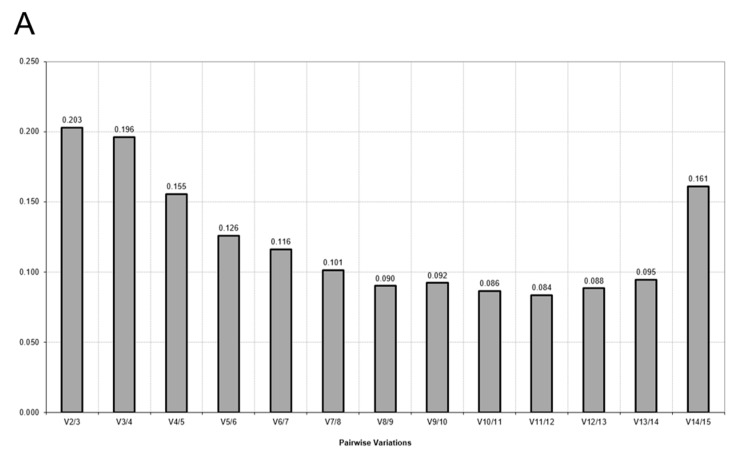

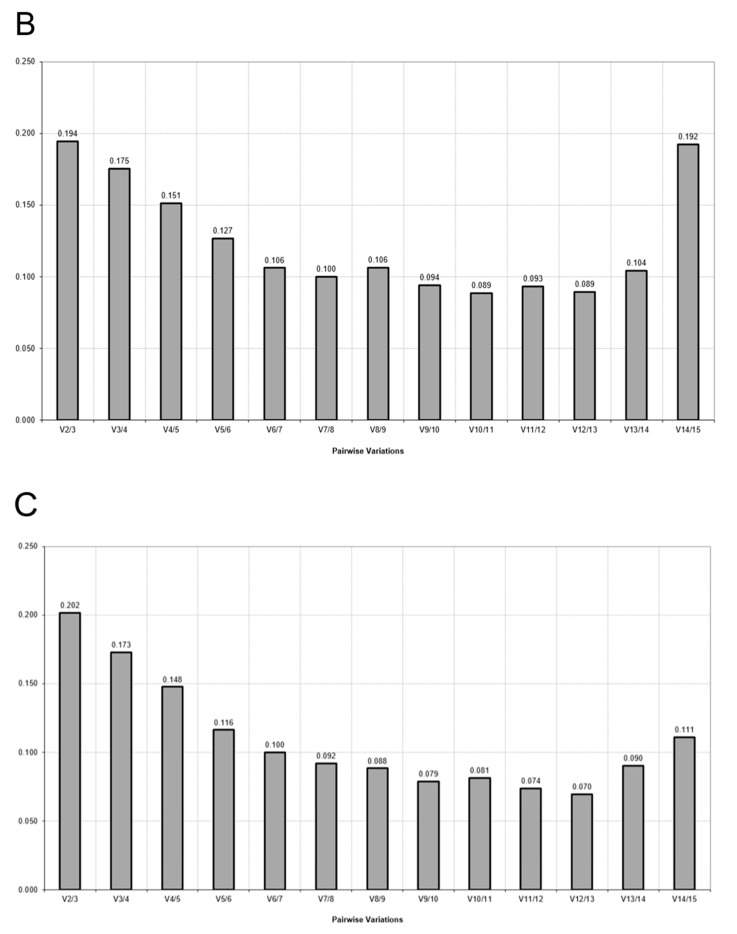
Determination of the optimal number of reference for normalization performed by pairwise variation analysis of candidate HKG under acid pH (**A**), physiological pH (**B**) and under both acid and physiological pH (**C**) culture conditions. A variation coefficient (*V*) below 0.15 indicates the optimal number of genes required for data normalization.

NormFinder identified *YWHAZ* as the most stable HKG, followed by *RPL13a* and *PPIA*. GeNorm confirmed *YWHAZ*, together with *18S rRNA*, to be the most stable genes. According to BestKeeper, the most stable genes were *GAPDH* and *18S rRNA*. The BestKeeper analysis also indicated that *GUSB*, *HMBS*, *SDHA*, *TUBB*, *HPRT1*, *G6PD*, and *TBP* exceeded the cut-off value of SD > 1.0. These genes should thus be avoided to normalize RT-qPCR data under acidic pH culture conditions. The Δ*C*_t_ analysis confirmed *YWHAZ* and *18S rRNA* as the most stable genes, but also recommended *GUSB*, which is one of the genes that should be avoid according to BestKeeper analysis. The coefficient of variation analysis confirmed *GAPDH* and *YWHAZ* to be the most stable HKG. The results of the pairwise variation calculation performed by GeNorm showed that the cutoff value of 0.15 was reached with 4 genes (*V*_4/5_ = 0.155), which indicated that 4 reference genes were required for accurate normalization (Figure 2A). Thus, according to the comprehensive ranking, we recommend *YWHAZ*, *GAPDH*, *18s rRNA* and *RPL13a* for normalization of gene expression under acidic pH culture conditions. On the contrary, the use of *TBP*, *G6PD*, and *SDHA* is not recommended since they are highly unstable HKG when cultured at pH 6.5.

We then considered the gene expression of osteosarcoma cell lines cultured at physiological pH (pH 7.4; Table 4).

NormFinder and the comparative Δ*C*_t_ method identified *YWHAZ* and *TUBB* as the most stable HKG. The GeNorm statistic algorithm indicated *RPL13a* and *B2M* as the two best-ranked genes, followed by *YWHAZ*. BestKeeper identified *GAPDH*, and confirmed *RPL13a* to be the to be most stable genes. According to BestKeeper calculation, *GUSB*, *G6PD*, *PPIA*, *PGK1*, *B2M*, *SDHA*, *TUBB*, *HMBS*, *HPRT1*, *ACTB*, and *TBP* exceeded the cutoff value (SD > 1.0). The coefficient of variation indicated that *GUSB* and *GAPDH* were the most stable HKG. The GeNorm analysis of the pairwise variation calculation *V* suggested that the optimal number of reference genes was 4 (*V*_4/5_ = 0.151; Figure 2B). Accordingly, the optimal normalization factor should be calculated as the geometric mean of *YWHAZ*, *RPL13a*, *GUSB* and *GAPDH*. Also in this case, *TBP* was confirmed the worse HKG.

Finally, we analyzed gene expression of HKG under both acidic and physiological pH culture conditions (pH 6.5 and 7.4; Table 5).

NormFinder confirmed that *YWHAZ* and *18S rRNA* were the most stable HKG. GeNorm identified *GUSB* and *HMBS* as other stable candidate genes. *18S rRNA* was one of the top ranked genes also in BestKeeper analysis, preceded only by *GAPDH*. The Δ*C*_t_ method confirmed *YWHAZ* and *GUSB* as the two best-ranked genes, followed by *18S rRNA*. The coefficient of variation confirmed *GAPDH* and *GUSB*, previously identified by the BestKeeper and Δ*C*_t_ methods, respectively. According to the variation coefficient *V*, the optimal normalization factor should be calculated as the geometric mean of 4 HKG (*V*_4/5_ = 0.148; Figure 2C). The comprehensive ranking of stability indicated that the top ranked genes are *YWHAZ*, *GUSB*, *GAPDH*, and *18S rRNA*, therefore the normalization factor should be calculated as the geometric mean of these HKG. Once more, *TBP* was confirmed as the less stable gene. To validate the data we obtained, we analyzed the expression of *c-MET*, a gene that has been often associated with osteosarcoma [27]. We found that the standard error (SE) of the expression of *c-MET* at pH 6.5 was significantly higher when we used *ACTB* (224.09 ± 207.86) or *TBP* (3.69 ± 2.04) for normalization in respect to the SE that we obtained by using the geometric mean of the 4 top ranked HKG (0.0025 ± 0.0010) (*p* < 0.05 for both *ACTB* or *TBP* vs. the geometric mean of the 4 selected HKG, *n* = 3, Figure S1).

## 3. Discussion

Tumor acidosis results from increased proton production determined by metabolic reprogramming toward up-regulation of glycolysis and tumor hypoxia caused by inadequate vascularization of the tumor bulk [2]. Tumor acidosis causes additional stress that fosters different aggressive phenotypes of cancer cells, including genomic instability [28], in turn regulating adaptation of gene expression [29]. Acid-induced alterations might also involve modifications in the expression of genes that are commonly used as a reference for RT-qPCR analysis, and it is therefore crucial to identify and validate stable HKG for accurate analysis.

In this study, we aimed to validate the most stable HKG among 15 candidate reference genes for the robust normalization of expression data of RT-qPCR analysis. The candidate HKG were previously selected as the most suitable genes for studying sarcoma cells, through a literature survey on the reference genes that have been used for the normalization of RT-qPCR data from tumors of mesenchymal origin [20,30]. The probability to include in the analyses co-regulated genes was avoided by exclusively selecting those HKG that belong to different functional classes and pathways [20]. Furthermore, the error due to RT-qPCR amplification efficiency was reduced by using primers with a uniform annealing temperature and an amplicon size of less than 150 bp [31].

We mimicked interstitial acidosis by using in vitro cell culture medium buffered at pH 6.5 [13]. Using deep sequencing, we verified that the selected HKG were expressed at basal levels in all the osteosarcoma cell lines both under acidic and physiological pH. This preliminary analysis demonstrated that the expression profiles of the selected genes were quite stable, with only slight fluctuation of the expression of some genes such as *TBP* or *TUBB*, which were then identified as unstable genes in acidic conditions. However, the analysis of the expression pattern of the selected HKG to assess their suitability as a reference for RT-qPCR experiments illuminated a different scenario. We applied different evaluation methods assessing gene stability to minimize errors associated with the application of one single software of evaluation, and to avoid the selection of co-regulated transcripts. Among these, NormFinder uses an ANOVA-based algorithm [23] to calculate the overall variation of the candidate reference genes in all samples, and also the variation of intra- and inter-groups. NormFinder assigns a stability value to each candidate gene using a model-based approach. Lower output scores indicate reduced variation of the expression of reference genes. GeNorm applies a pairwise comparison method based on the calculation of the expression stability score (M) [24]. The lower the M value, the more stable the expression of the reference gene, with values of M that surpass the cutoff value of 1.5 not considered stable across the examined conditions. GeNorm ranks genes on the basis of their M value, performing stepwise exclusion of the gene with the highest M-value (the least stable expressed gene), and recalculating the M-values of the remaining genes. BestKeeper is a basic descriptive statistic method of evaluation, which calculates gene stability on the basis of pairwise correlation analysis of all pairs of candidate reference genes [25]. This means that the geometric mean of the *C*_t_ values of the candidate reference genes is compared with standard deviation (SD) and stability value (SV); lower index scores represent stable reference genes. The values that surpass the cutoff value of SD > 1.0 are considered unstable. The Δ*C*_t_ method provides the most stably expressed reference gene based on Δ*C*_t_ value variation [26] by comparing the relative transcription of pairs of gene. The stability of candidate HKG is ranked according to repeatability among all samples. Rank order is determined based on mean Δ*C*_t_ values; the lower the average SD, the more stable the reference gene. The coefficient of variation estimates the SD over the average of a random variable [20]. Gene stability was calculated by dividing the standard deviation (SD) of *C*_t_ by the mean *C*_t_ value. Moreover, we calculated the minimal number of genes required for adequate normalization of RT-qPCR data. GeNorm determines the number of control genes require for accurate normalization performing the pairwise variation (*V_n_*_/*n*+1_) analysis between the normalization factors NF*_n_* and NF*_n_*_+1_ for each gene analyzed [24]. *V* values below the cutoff value 0.15 indicated the optimal number of genes required for data normalization. To overcome the discrepancies and obtain a final rank, we calculated the comprehensive HKG ranking by considering the lowest value of the geometric average of the rank obtained from all the algorithms and methods of stability calculation [20].

First, our analyses suggested that the expression stability of most of the HKG is highly influenced by pH and that four HKG are needed for accurate evaluation of RT-qPCR data. These findings underline that, for gene expression analyses of tumor cells maintained under low pH conditions, a universal internal control based on only one ideal HKG may produce inconsistent data, thus we recommend to normalize the gene of interest with a panel of HKG whose expression has been proven to be minimally variable and most robust in the specific condition investigated. Moreover, according to our analyses, to evaluate gene expression under acid conditions, we suggest calculating the normalization factor from the geometric mean of *C*_t_ of *YWHAZ*, *RPL13a*, *GUSB*, and *GAPDH*, whereas under physiological pH the normalization factor should be calculated from the geometric average expression of *YWHAZ*, *RPL13a*, *GAPDH*, and *18S rRNA*. Most importantly, to compare gene expression under acidic and physiological pH, the optimal normalization factor should derive from the geometric mean of *YWHAZ*, *GAPDH*, *GUSB*, and *18S rRNA*. Notably, *YWHAZ* and *GAPDH* were revealed as the most stable HKG in all the pH conditions, confirming its suitability as a HKG to gene normalization of sarcoma cells [20]. The analyses also suggested that the use of *TPB*, as well as other commonly used housekeepers such as *ACTB*, *B2M*, and *TUBB* [32,33], should be avoided.

This work is the first validation of reference genes in acidic pH, and provides useful information to perform future gene expression studies in osteosarcoma. Furthermore, the protocol that we set up for osteosarcoma cell lines to identify the best set of HKG in acid conditions can be used for the future for other tumor histotypes.

## 4. Materials and Methods

### 4.1. Cell Cultures

Osteosarcoma cell lines MG-63, HOS, Saos-2 were purchased from American Type Culture Collection (ATCC, Manassas, VA, USA), and cultured in Iscove’s modified Dulbecco’s medium (IMDM, Gibco, Carlsbad, CA, USA), plus 20 U/mL penicillin, 100 mg/mL streptomycin, and 10% heat-inactivated fetal bovine serum (FBS) (complete IMDM) at 37 °C in a humidified 5% CO_2_ atmosphere. For assays with different pH, cells were seeded in complete medium, and after 24 h media were changed. New media were set at a specific pH by using different concentrations of sodium bicarbonate to preset pH in 5% CO_2_ atmosphere, according to the Henderson-Hasselbach equation [13]. At the end-point of each experiment, the final pH in the supernatant was always measured by a digital pH-meter (pH 301, HANNA Instruments, Woonsocket, RI, USA).

### 4.2. Illumina Genome Analyzer Sequencing and Data Analysis

In order to select a panel of stable HKG for RT-qPCR analysis, a deep sequencing analysis of MG-63, HOS, and Saos-2 osteosarcoma cell models was performed to compare the global transcriptional expression of osteosarcoma cells under acidic and physiological conditions. Briefly, total RNA was collected from the cell lysate in acid guanidinium thiocyanate-phenol-chloroform [34]. The total RNA was quantified by Bioanalyzer (Agilent, Santa Clara, CA, USA) following the manufacturer’s instructions. RIN (RNA Integrity Number) and A260/A280 ratio of the prepared total RNA were all 10, and over 1.8, respectively. The library of template molecules for high throughput DNA sequencing was converted from the total RNA using TruSeq RNA Sample Prep Kit v2 (Illumina, San Diego, CA, USA), following the manufacturer’s protocol. The library was also quantified with Bioanalyzer (Agilent), following the manufacturer’s instruction. The library (7 pM) was subjected to cluster amplification on a Single Read Flow Cell v4 with a cluster generation instrument (Illumina). Sequencing was performed on a Genome Analyzer GAIIx for 70 cycles using Cycle Sequencing v4 regents (Illumina). Human genome build 19 (hg19) were downloaded from University of California, Santa Cruz genome browser (http://genome.ucsc.edu/). Image analysis and base calling were performed using Off-Line Basecaller Software 1.6 (Illumina). Reads were aligned using ELAND v2 of CASAVA Software 1.7 with the sequence data sets. Transcript coverage for every gene locus was calculated from the total number passing filter reads that mapped, by ELAND-RNA, to exons. These analyses were performed using default parameters. The data were viewed using Genome Studio Software (Illumina). The advanced analysis for quantification with Quantile normalization algorithm was performed using Avadis NGS software (version 1.5, Strand Scientific Intelligence Inc., San Francisco, CA, USA). The filtering was per-formed using default parameters. All new data has been deposited in DDBJ/EMBL/GenBank under DRA004087 and DRA004091.

### 4.3. RNA Isolation and cDNA Synthesis

Total RNA was extracted with NucleoSpin RNA II (Macherey-Nagel, Düren, Germany) from each biological sample according to the manufacturer’s instructions (on-column genomic DNA digestion was performed as per said instructions), and RNA concentration and the absorbance ratio A_260/280_ were measured by spectrophotometer Nanodrop Spectrophotometer (NanoDrop Technologies, Wilmington, DE, USA). Total RNA (0.7 μg) were reverse-transcribed into cDNA in 20 μL final volume, using MuLV Reverse Transcriptase and RNase inhibitor (Applied Biosystems, Foster City, CA, USA). First-strand cDNA was synthesized using random hexamers. For each sample, 3 biological replicates were processed.

### 4.4. RT-qPCR

RT-qPCR was performed by using a Light Cycler instrument and the Universal Probe Library system (Roche Applied Science, Monza, Italy). Probe and primers were selected by using a web-based assay design software (ProbeFinder https://www.roche-applied-science.com), and were further controlled using Oligo Primer Analysis Software, v. 7 (Molecular Biology Insights, Inc., Cascade, CO, USA). Only primers spanning an exon–exon junction and producing a PCR amplificate with length between 70 and 150 base pairs were selected. All the primers designed were analyzed by BLAST to verify their specificity (National Center for Biotechnology Information). All cDNA were diluted 1:10, and 10 μL were used as template and included in a 20 μL of total volume of RT-qPCR reaction. The protocol of amplification was: 95 °C for 10 min; 95 °C for 10 s, 60 °C for 30 s, and 72 °C for 1 s for 45 cycles; 40 °C for 30 s. *c-MET* expression (NM_001127500) was evaluated using the following primers: fwd 5′-cagagacttggctgcaagaa-3′, rev 5′-ggcaagaccaaaatcagca-3′. The relative expression of *c-MET* was normalized for the reference gene *TBP* or *ACTB* or for the geometric average of *YWHAZ*, *GUSB*, *GAPDH* and *18S rRNA*. The relative expression of *c-MET* was calculated using the ΔΔ*C*_t_ model [20]. Each assay included a blank.

### 4.5. Stability and Statistical Analysis for Reference Genes

We used 4 algorithms to determine the stability of the candidate HKG, beside the calculation of coefficient of variation of candidate HKG [20]. The 4 algorithms used are NormFinder [23], geNorm [24], Δ*C*_t_ method [26], and BestKeeper [25]. We used GeNorm also to calculate the minimal number of genes required for adequate normalization of RT-qPCR data. For NormFinder and GeNorm analyses, *C*_t_ values obtained from RT-qPCR analyses were converted to linear scale by comparative *C*_t_ method, using the lowest *C*_t_ value as calibrator. These linear relative quantities were used as input data for further analysis of gene stability. BestKeeper, Δ*C*_t_ method, and the coefficient of variation directly utilize the *C*_t_ value obtained from RT-qPCR analyses to calculate gene stability. Results were reported as mean of *C*_t_ values ± standard error of mean (SE). Standard deviation (SD) of Δ*C*_t_ values was calculated as pooled standard deviation (SDpooled). Results are representative of 3 biological replicates.

### 4.6. Comprehensive Analysis of Ranks

The analyses performed by NormFinder, geNorm, BestKeeper, Δ*C*_t_, and coefficient of variation method showed some differences in the stability rank of the HKG. The net final ranking was obtained considering the lowest value of the geometric average of the ranks [20] obtained by the above-described methods.

### 4.7. Statistical Analysis

Statistical analysis was performed with the GraphPad Prism 7.04 software (GraphPad Software Inc, La Jolla, CA, USA). Data were expressed as mean ± SE. One-tailed Mann Whitney *U* test was used to analyses the difference between the SE of two different groups. Only *p* values < 0.05 were considered for statistical significance.

## 5. Conclusions

A large number of studies have investigated the validation of reference genes in many different tissues and cell types. However, different microenvironmental conditions might alter the expression of HKG, thereby affecting the interpretation of gene expression data of cancer cells. To the best of our knowledge, this is the first study addressing the validation of reliable HKG in cells maintained in an acidic microenvironment. Stability analyses revealed that, to obtain reliable results in osteosarcoma, at least 4 HKG should be considered. Moreover, by using different algorithms, we identified *YWHAZ*, *GAPDH*, *GUSB*, and *18S rRNA* as the most stable HKG to study the molecular alterations that occur and that are induced after acid stress. For the future, our experimental approach can be used for studying gene expression under acid conditions also for other tumor histotypes.

## Figures and Tables

**Table 1:** Candidate housekeeping genes (HKG). Gene symbol, name, accession number, primer forward and reverse sequences, and amplicon size are shown.

Symbol	Gene Name	Accession No.	Forward Primer 5′-3′	Reverse Primer 5′-3′	Amplicon Size (nt)
*18S rRNA*	18S ribosomal RNA	X03205.1	gcaattattccccatgaacg	gggacttaatcaacgcaagc	68
*ACTB*	Actin β	NM_001101.2	ccaccgcgagaagatga	ccagaggcgtacagggatag	97
*B2M*	β-2-Microglobulin	NM_004048.2	ttctggcctggaggctatc	tcaggaaatttgactttccattc	86
*G6PD*	Glucose-6-phosphate dehydrogenase	M24470.1|M24470	gaagggccacatcatctctg	atctgctccagttccaaagg	76
*GAPDH*	Glyceraldehyde 3-phosphate dehydrogenase	NM_002046.3	agccacatcgctcagacac	gcccaatacgaccaaatcc	66
*GUSB*	β-Glucuronidase	M15182.1|M15182	cgccctgcctatctgtattc	tccccacagggagtgtgtag	91
*HMBS*	Hydroxymethylbilane synthase	NM_000190.3	tgtggtgggaaccagctc	tgttgaggtttccccgaat	92
*HPRT1*	Hypoxanthine phosphoribosyltransferase 1	M31642.1|M31642	tgaccttgatttattttgcatacc	cgagcaagacgttcagtcct	102
*PGK1*	Phosphoglycerate kinase 1	NM_000291.3	ggagaacctccgctttcat	gctggctcggctttaacc	78
*PPIA*	Peptidylprolyl isomerase A	NM_021130.3	atgctggacccaacacaaat	tctttcactttgccaaacacc	97
*RPL13a*	Ribosomal protein L13a	NM_012423.3	caagcggatgaacaccaac	tgtggggcagcatacctc	95
*SDHA*	Succinate dehydrogenase complex, subunit A	NM_004168.2	ggacctggttgtctttggtc	ccagcgtttggtttaattgg	93
*TBP*	TATA-binding protein	NM_001172085.1	ttgggttttccagctaagttct	ccaggaaataactctggctca	140
*TUBB*	Tubulin, β class I	NM_178014.2	ataccttgaggcgagcaaaa	tcactgatcacctcccagaac	113
*YWHAZ*	Tyrosine 3-monooxygenase/tryptophan 5-monooxygenase activation protein zeta polypeptide	NM_003406.3	ccgttacttggctgaggttg	tgcttgttgtgactgatcgac	67

**Table 2:** Raw *C*_t_ values of the candidate HKG in acid and physiological conditions (pH 6.5 and 7.4, respectively).

Gene	*C*_t_ Value at pH 6.5 and 7.4 (Mean ± SD)	*C*_t_ Value at pH 6.5 (Mean ± SD)	*C*_t_ Value at pH 7.4 (Mean ± SD)	Δ*C*_t_ Value (Difference of Mean ± SD Pooled)
*18S rRNA*	9.64 ± 0.91	9.48 ± 0.80	9.79 ± 1.04	−0.32 ± 0.18
*ACTB*	20.47 ± 1.38	20.35 ± 1.10	20.58 ± 1.68	−0.24 ± 0.30
*B2M*	20.75 ± 1.25	20.15 ± 0.89	21.35 ± 1.30	−1.20 ± 0.49
*G6PD*	30.39 ± 1.55	30.48 ± 1.76	30.31 ± 1.39	0.17 ± 0.21
*GAPDH*	20.79 ± 0.77	20.95 ± 0.71	20.63 ± 0.84	0.32 ± 0.15
*GUSB*	30.23 ± 1.27	30.07 ± 1.41	30.39 ± 1.16	−0.33 ± 0.21
*HMBS*	27.34 ± 1.41	27.54 ± 1.43	27.14 ± 1.45	0.40 ± 0.29
*HPRT1*	26.95 ± 1.60	26.93 ± 1.75	26.96 ± 1.54	−0.04 ± 0.21
*PGK1*	22.41 ± 1.33	22.43 ± 1.39	22.39 ± 1.35	0.04 ± 0.16
*PPIA*	21.08 ± 1.35	21.41 ± 1.25	20.75 ± 1.43	0.66 ± 0.32
*RPL13a*	20.12 ± 1.02	20.28 ± 1.05	19.97 ± 1.03	0.30 ± 0.18
*SDHA*	25.04 ± 1.58	24.84 ± 1.84	25.24 ± 1.36	−0.40 ± 0.27
*TBP*	27.59 ± 2.68	26.87 ± 3.06	28.31 ± 2.19	−1.43 ± 0.81
*TUBB*	22.72 ± 1.59	23.22 ± 1.65	22.23 ± 1.44	1.00 ± 0.46
*YWHAZ*	23.12 ± 1.07	23.39 ± 0.94	22.85 ± 1.17	0.54 ± 0.27

**Table 3:** Ranking of the expression of candidate HKG under acid pH culture conditions (pH 6.5).

Gene	NormFinder		GeNorm		BestKeeper		Δ*C*_t_		Coefficient of Variation	
	Stability Value	Rank	M Value	Rank	ST.DEV [+/− CP]	Rank	ST.DEV	Rank	CV	Rank
*YWHAZ*	0.265	1	0.518	1	0.776	4	1.076	1	0.040	2
*RPL13a*	0.389	2	0.756	5	0.924	6	1.203	5	0.052	6
*PPIA*	0.398	3	0.682	4	0.969	7	1.164	4	0.058	9
*18S rRNA*	0.401	4	0.518	1	0.604	2	1.144	3	0.084	14
*GUSB*	0.431	5	0.802	6	1.166	9	1.142	2	0.047	4
*ACTB*	0.461	6	0.861	8	0.872	5	1.330	10	0.054	7
*HMBS*	0.471	7	0.826	7	1.242	10	1.205	6	0.052	5
*GAPDH*	0.519	8	0.589	3	0.537	1	1.260	7	0.034	1
*PGK1*	0.533	9	0.991	11	0.988	8	1.276	8	0.062	10
*HPRT1*	0.570	10	0.911	9	1.397	13	1.287	9	0.065	11
*TUBB*	0.578	11	0.953	10	1.345	12	1.332	11	0.071	12
*SDHA*	0.655	12	1.085	13	1.338	11	1.461	12	0.074	13
*B2M*	0.697	13	1.039	12	0.680	3	1.467	13	0.044	3
*G6PD*	0.874	14	1.162	14	1.493	14	1.757	14	0.058	8
*TBP*	1.205	15	1.406	15	2.223	15	2.991	15	0.114	15

**Table 4:** Ranking of the expression of candidate HKG under physiological pH culture conditions (pH 7.4).

Gene	NormFinder		GeNorm		BestKeeper		Δ*C*_t_		Coefficient of Variation	
	Stability Value	Rank	M Value	Rank	ST.DEV [+/− CP]	Rank	ST.DEV	Rank	CV	Rank
*YWHAZ*	0.329	1	0.660	3	0.885	4	0.938	1	0.051	4
*TUBB*	0.336	2	0. 836	8	1.109	11	0.967	2	0.065	11
*ACTB*	0.413	3	0.771	5	1.298	14	0.994	3	0.082	14
*PPIA*	0.444	4	0.716	4	1.018	7	1.032	4	0.069	12
*SDHA*	0.458	5	0.914	11	1.081	10	1.043	6	0.054	7
*RPL13a*	0.462	6	0.620	1/2	0.670	2	1.072	9	0.052	5
*GUSB*	0.469	7	0.815	7	1.002	5	1.047	7	0.038	1
*HPRT1*	0.493	8	0.796	6	1.261	13	1.042	5	0.057	8
*18S rRNA*	0.494	9	0.882	10	0.828	3	1.079	10	0.106	15
*B2M*	0.509	10	0.620	2/1	1.038	9	1.047	8	0.061	10
*PGK1*	0.510	11	0.939	12	1.034	8	1.118	12	0.060	9
*HMBS*	0.520	12	0.962	13	1.169	12	1.184	13	0.053	6
*GAPDH*	0.557	13	0.862	9	0.658	1	1.117	11	0.040	2
*G6PD*	0.691	14	1.026	14	1.015	6	1.438	14	0.046	3
*TBP*	0.748	15	1.130	15	1.362	15	1.805	15	0.077	13

**Table 5:** Ranking of the expression of candidate HKG under both acidic and physiological pH culture conditions (pH 6.5 and 7.4).

Gene	NormFinder		GeNorm		BestKeeper		Δ*C*_t_		Coefficient of Variation	
	Stability Value	Rank	M Value	Rank	ST.DEV [+/− CP]	Rank	ST.DEV	Rank	CV	Rank
*YWHAZ*	0.335	1	0.861	6	0.880	4	1.072	1	0.046	3
*18S rRNA*	0.441	2	0.914	8	0.719	2	1.158	3	0.095	14
*GUSB*	0.455	3	0.686	1/2	1.102	9	1.136	2	0.042	2
*PPIA*	0.463	4	0.787	4	1.032	7	1.171	5	0.064	11
*ACTB*	0.469	5	0.937	9	1.097	8	1.211	7	0.068	12
*RPL13a*	0.473	6	0.838	5	0.796	3	1.167	4	0.051	4
*PGK1*	0.528	7	1.006	11	1.011	6	1.218	8	0.059	8
*HPRT1*	0.535	8	0.694	3	1.329	13	1.189	6	0.059	7
*TUBB*	0.550	9	0.973	10	1.343	14	1.292	11	0.070	13
*GAPDH*	0.556	10	0.890	7	0.638	1	1.220	9	0.037	1
*HMBS*	0.565	11	0.686	1/2	1.228	10	1.226	10	0.051	6
*SDHA*	0.598	12	1.038	12	1.241	11	1.301	12	0.063	10
*B2M*	0.649	13	1.084	13	0.890	5	1.452	13	0.061	9
*G6PD*	0.794	14	1.144	14	1.264	12	1.598	14	0.051	5
*TBP*	0.965	15	1.330	15	1.683	15	2.537	15	0.097	15

## Supplementary Materials

The following are available online at http://www.mdpi.com/1422-0067/19/10/2930/s1.

## Abbreviations

18S rRNA	18S ribosomal RNA
ACTB	Actin β
B2M	β-2-Microglobulin
G6PD	Glucose-6-phosphate dehydrogenase
GAPDH	Glyceraldehyde 3-phosphate dehydrogenase
GUSB	β-Glucuronidase
HKG	Housekeeping genes
HMBS	Hydroxymethylbilane synthase
HPRT1	Hypoxanthine phosphoribosyltransferase 1
PGK1	Phosphoglycerate kinase 1
PPIA	Peptidylprolyl isomerase A
RPL13a	Ribosomal protein L13a
SDHA	Succinate dehydrogenase complex, subunit A
TBP	TATA-binding protein
TUBB	Tubulin, β class I
YWHAZ	Tyrosine 3-monooxygenase/tryptophan 5-monooxygenase activation protein zeta polypeptide
