# Supplementary material for: Validation of Suitable Housekeeping Genes for the Normalization of mRNA Expression for Studying Tumor Acidosis
Source: Int J Mol Sci. 2018 Sep 26;19(10):2930. doi: 10.3390/ijms19102930 (PMC6213411; doi:10.3390/ijms19102930)
Supplement: Supplementary file 1 [file ijms-19-02930-s001.pdf]

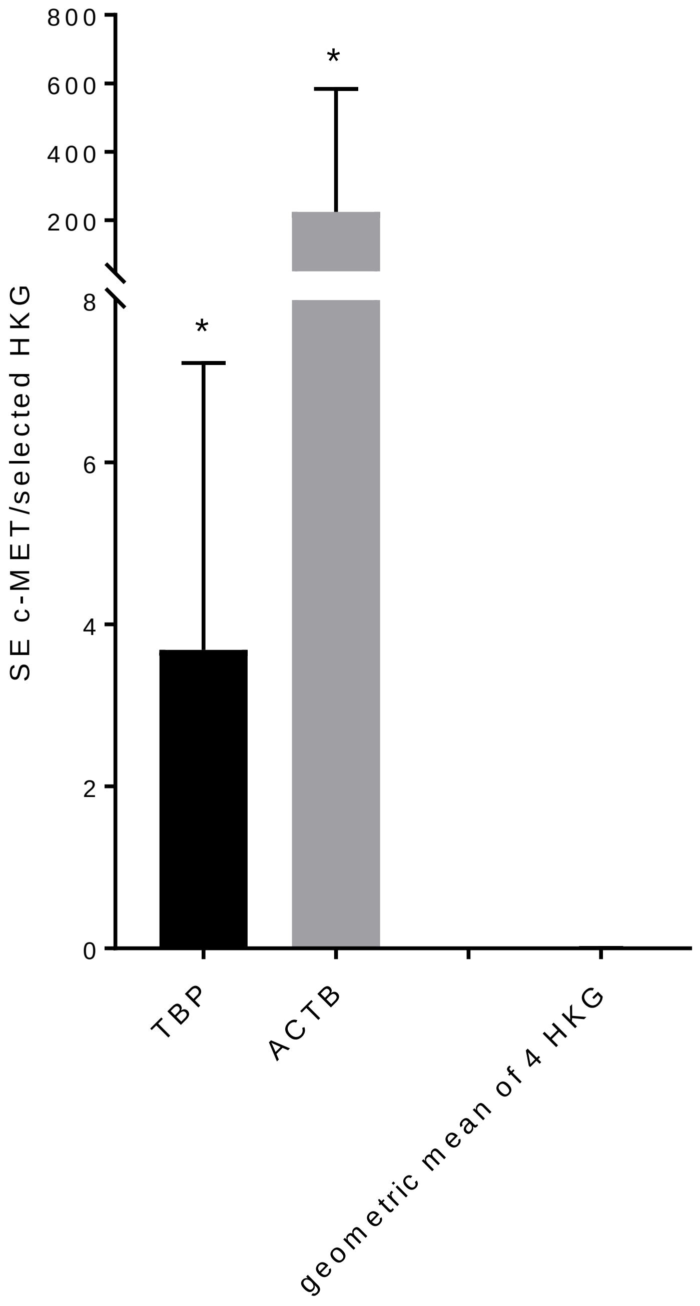

Figure S1. The SE of the expression of *c-MET* at pH 6.5. *c-MET* expression was normalised for *TBP* or *ACTB* or for the geometric average of *YWHAZ*, *GUSB*, *GAPDH* and *18S rRNA*. $p < 0.05$ for both ACTB or TBP vs the geometric mean of the 4 selected HKG, $n = 3$.
